# Genetic Polymorphism Study on* Aedes albopictus* of Different Geographical Regions Based on DNA Barcoding

**DOI:** 10.1155/2018/1501430

**Published:** 2018-05-29

**Authors:** Yiliang Fang, Jianqing Zhang, Rongquan Wu, Baohai Xue, Qianqian Qian, Bo Gao

**Affiliations:** ^1^Fujian International Travel Healthcare Center, Fuzhou, Fujian 350001, China; ^2^Quanzhou Entry-Exit Inspection and Quarantine Bureau Comprehensive Technical Service Center, Quanzhou 362000, Fujian Province, China; ^3^Fujian Medical University, Fuzhou, Fujian 350001, China

## Abstract

*Aedes albopictus* is a very important vector for pathogens of many infectious diseases including dengue fever. In this study, we explored the genetic polymorphism of* Aedes albopictus* strains in different geographical regions using DNA barcoding of mitochondrial COI (*MT-COI*) gene. We collected* MT-COI* sequence of 106* Aedes albopictus* mosquitos from 6 provinces in China including Fujian, Guangdong, Hainan, Yunnan, and Taiwan. The length of the sequences is 709bp with the content of A+T (67.7%) greater than that of G+C (32.3%). We identified mutations in 90 (13.68%) loci, of which 57 (63.33%) are transitions, 28 (31.11%) are transversions, and 5 (5.56%) are hypervariable loci. In addition, we obtained 42 haplotypes, 4 (9.52%) of which are shared among different populations. The haplotype diversity of* Aedes albopictus* is 0.882 and nucleotide diversity is 0.01017. Moreover, the pedigree network diagram shows that most haplotypes are under parallel evolution, suggesting a local expansion of* Aedes albopictus* in history. Finally, the Neighbor-Joining tree of* MT-COI* haplotypes reveals a certain correlation between haplotype clusters and geographical distribution, and there are differences among* Aedes albopictus *in different geographical regions. In conclusion, DNA barcoding of* MT-COI* gene is an effective method to study the genetic structure of* Aedes albopictus*.

## 1. Introduction

*Aedes albopictus*, belonging to the* Diptera Nematocera Culicidac Aedes* genus, are widely distributed all over the world [1–4]. It was originated in Southeast Asia and spread rapidly into many countries in Africa, the Middle East, Europe, and America in the past three decades [1, 5]. International trade, especially that of used tires at ports, has accelerated its transmission [6]. In China, though* Aedes albopictus *was observed from Hainan island in the south all over to Liaoning province in the north, it was most gathered mostly in the south of 30° north latitude such as the Fujian province [7]. Understanding the population genetic polymorphism of* Aedes albopictus* is critical to its prevention and control.

DNA barcoding is a biometrics based on the mitochondrial cytochrome C oxidase subunit I (*MT-COI*) gene (about 500-600 bp), which was first introduced by Canadian biologist Paul Hebert in 2003 [8, 9]. This technology has received widespread attention since then. Mitochondrial COI gene sequences with strict maternal inheritance, conservative genetic makeup, and moderate evolution rate but higher nuclear DNA characteristics [10] have been widely used in the identification of mammals, fish, insects, birds, and other species [11–15]. However, to our best knowledge, there is no research on genetic polymorphism of* Aedes albopictus* at species level based on DNA barcoding at present. The reason might be due to the high cost in collecting and sequencing* Aedes albopictus *strains in a wide region, which might even be infeasible in a small country.

In this study, we collected and sequenced the* MT-COI* gene of 106* Aedes albopictus* strains from 6 provinces in China including Fujian, Guangdong, Hainan, Yunnan, and Taiwan. We then performed down-stream bioinformatics and phylogenetic analysis on the sequences. This study reveals the genetic polymorphism of* Aedes albopictus *in South China, which is important for the prevention and control of infectious diseases spread by* Aedes albopictus *like dengue fever and yellow fever [16].

## 2. Materials and Methods

### 2.1. Experimental Mosquitos

**Figure 1:**
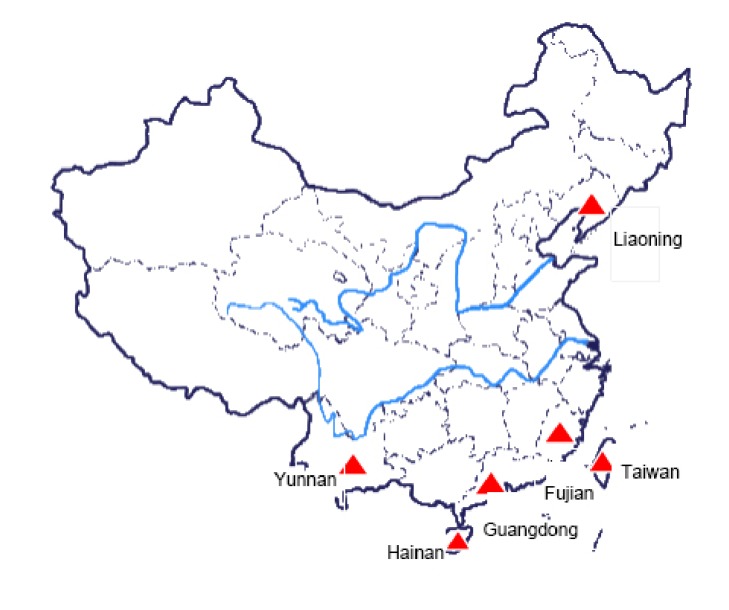
Collecting places of different geographical strains of* Aedes albopictus*.

We collected mosquitos from Fujian province (Fuzhou, Zhangzhou, Jiangling, and Wuyishan), Guangdong province (Guangzhou), Hainan province (Diaoluoshan, Maoyang), Yunnan province (Mengla), Liaoning province (Xishan), and Taiwan (Taipei, Kaohsiung) (Figure 1).

### 2.2. Mosquito DNA Preparation and Amplification

Nucleic acid was extracted using the TIANamp Genomic DNA Kit after removing the head of mosquitos. We then used a pair of universal primers including LCO1490 (5′-GGTCAACAAATCATAAAGATATTGG-3′) and HCO2198 (5′-TAAACTTCAGGGTGACCAAAAAAT-CA-3′) to amplify them [17]. The reaction system was set as follows: Ex Taq TaKaRa 0.25*μ*L, 10XEx Taq Buffer 5*μ*L, dNTP Mixture 4*μ*L, Template DNA 3*μ*L (if the light belt can be properly increased), and upper and lower primer (10 *μ*M) with each 1*μ*L, making a total volume of 50*μ*L by adding ddH_2_O. In addition, the amplification conditions are set as follows: predenaturation at 94°C for 3 min, denaturation at 94°C for 30 s, annealing at 55°C for 30 s, extension at 72°C for 45 sec, a total of 30 cycles, and final elongation at 72°C for 5 min.

### 2.3. PCR Product Purification and Cloning

The PCR products were cloned by Universal DNA Purification kit, equipped with LB liquid medium, LB solid medium, and 50 mg/ml IPTG. The PCR products were digested with PCR Identification Kit for Recombinant pGM-T Clone, which were sent out for sequencing.

### 2.4. Data Analysis

*Sequence Verification and Alignment. *By referring to the NCBI sequence database, homology search was performed against existing COI gene sequences of* Aedes albopictus* in GenBank, and sequences with identity greater than or equal to 98% were selected [18].

*Sequence Analysis. *The aligned sequences were input into the multiple sequence alignment tool Clustal X (v1.8) for multiple sequence alignment [19, 20]. The MEGA6 software package was used to summarize the statistics of sequence characteristics, including base content, the number of transversions, and transitions, and calculate the genetic distance of the sequences [21]. Taking* Chironomidae nepeanensis* (GenBank: KC750313.1) as the outgroup, we constructed Neighbor-Joining (NJ) tree and Maximum Likelihood (ML) tree according to the genetic distance Kimura 2-parameter method and bootstrapped 1000 times to test the reliability of the branch trees [21–23]. The DnaSP 5.0 software [24] was used to identify the polymorphism sites and calculate the haplotype diversity and nucleotide diversity of each geographical population of* Aedes albopictus*. The median joining method of Network4.6 software was used to construct haplotype pedigree network diagram. The analysis of molecular variance (AMOVA v3.1) was used to calculate the genetic differentiation within and between populations, including Fst (F-statistics) and Nm (Nm = (L − Fst)/4Fst) [25]. Arlequin was used to do mismatch analysis and neutrality test. We then calculated sum of squared deviation (SSD) and Harpending's Raggedness (HR) indices through mismatch analysis and constructed observation simulation model [26, 27], drew bifurcation point distribution, and explored the evolutionary history of* Aedes albopictus* population. Using the Tajima's D [28] and Fu's Fs values [29] of the neutrality test, we further explored the population expansion mechanism.

The ancestral population size (*θ*) is calculated as follows: *θ* = 2Nu, where N represents the effective number of female mosquitos in the population, u represents the mutation rate per generation, and u = *μ*k (*μ* is the mutation rate per point per generation and k is the base number of the analyzed sequences). We used *τ* = 2ut to estimate the approximate generation number (t) of population expansion [26] and set the mutation rate per point per generation of* Aedes albopictus* which is 1 × 10^−8^ [30].

Mantel test was performed by online software IBD Web Service (IBDWS) [31] to evaluate the correlation between genetic differentiation coefficient and geographical distance.

## 3. Results

### 3.1. Sequence Features

The mitochondrial COI gene was obtained with length 709bp. According to the sequence alignment of* Aedes albopictus* in NCBI database, the sequence identity was 99%-100% showing very small difference among the 106 sequences.

After sequence alignment, we took the 658 bp fragment (with the universal primer removed) for subsequent analysis. The overall base composition of the fragment is A (28.5%), T (39.2%), C (16.7%), and G (15.6%). The A+T content (67.7%) is greater than that of G+C (32.3%). There were 90 (13.68%) polymorphism sites, of which 57 (63.33%) are transitions, 28 (31.11%) are transversions, and 5 (5.56%) are hypervariable loci (Table S1).

### 3.2. The Relationships among Haplotypes of Different Geographical Strains

We obtained 42 haplotypes on 6 geographical strains of* Aedes albopictus MT-COI* gene (Table 1), 4 (9.52%) of which are shared haplotypes among different populations. They are h2, h6, h18, and h19, respectively: h2 is shared by Fujian, Guangdong, Taiwan, and Liaoning, h6 is shared by Fujian and Guangdong, and h18 and h19 are shared by Hainan and Yunnan. The result indicates that there are gene exchanges among the populations. However, it can be seen from the exclusive haplotypes that* Aedes albopictus* has some genetic differentiation. Haplotype diversity (Hd) and nucleotide diversity (Pi) were used to indicate the degree of haplotype differentiation and the degree of nucleotide sequence variation. The haplotype diversity of* Aedes albopictus* is 0.882 and nucleotide diversity is 0.01017. The haplotype diversity of geographical strains is ordered as Yunnan > Fujian > Hainan > Taiwan > Guangdong > Liaoning strains. The nucleotide diversity is ordered as Fujian > Yunnan > Hainan > Taiwan > Guangdong > Liaoning strains. Fujian strains had the largest number of polymorphism sites (s = 66) and haplotype number (n = 12) and thus also had the largest nucleotide diversity (Pi = 0.03316). Its haplotype diversity is 0.87895, only slightly smaller than that of Yunnan strains (Hd = 0.88421). There were no differences between the 5 individuals in Liaoning populations.

**Figure 2:**
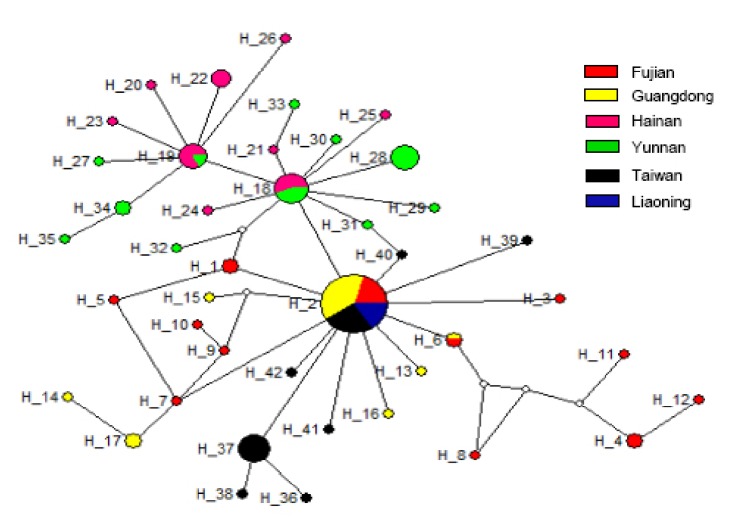
The haplotypes pedigree network diagram of different geographical strains of* Aedes albopictus MT-COI* gene.

The median joining method in software Network4.6 was used to construct haplotypes pedigree network of 42 haplotypes from 6 geographical strains of* Aedes albopictus* mtDNA-COI gene (Figure 2). In the network, each circle represents a haplotype, the size of the circle represents the number of homozygous haplotypes, different colors indicate different geographical haplotypes, and the blank circles indicate undetected haplotypes. As can be seen from the network, there is a certain level of parallel evolution between the haplotypes, suggesting the expansion of* Aedes albopictus* in history. Among them, unchecked haplotypes are rare (5), but shared haplotypes H2 (34, 32.08%), H18 (9, 8.49%), and H19 (6, 5.66%) are highly distributed in the population, which may be the sources of expansion. It can be seen that Yunnan + Hainan strains and other geographical strains are roughly divided into two categories, but there are individual haplotypes intersecting with each other.

**Figure 3:**
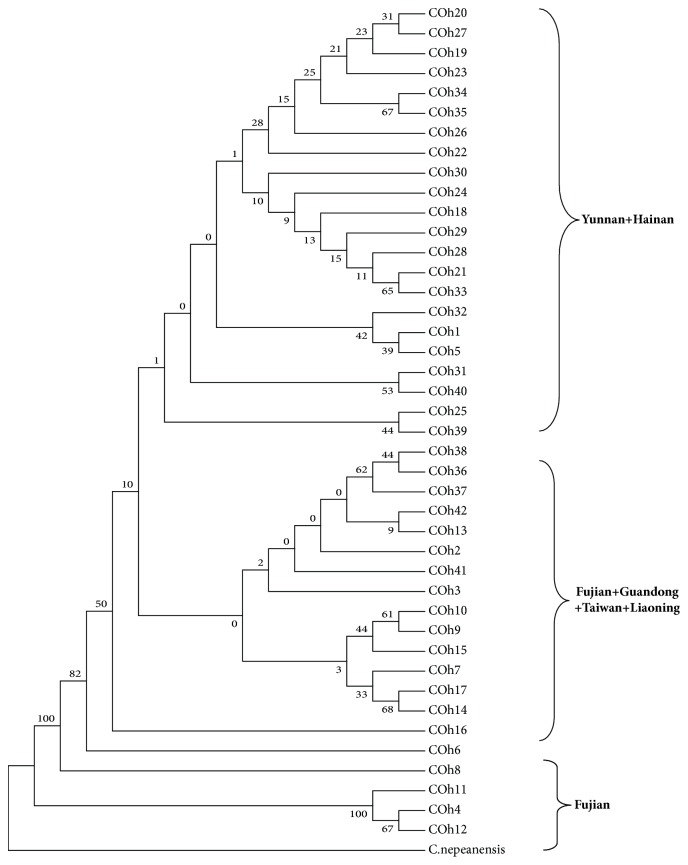
The NJ tree of* Aedes albopictus MT-COI* gene haplotypes from different geographical strains.

MEGA6 was used to calculate the genetic distances among* MT-COI* gene haplotypes of different geographical strains and the NJ and ML methods were used to construct the phylogenetic trees respectively with 1,000 times bootstrapping to assess branch confidence. The trees constructed from the two methods are quite similar, so we took NJ tree for illustration (Figure 3). As can be seen, part of Fujian haplotypes was clustered into one branch (H4, H11, and H12) with high confidence and the rest (Hainan + Yunnan) (Guangdong + Taiwan + Liaoning + part of Fujian) were clustered into their respective branches with low confidence. The NJ tree roughly coincides with the haplotypes pedigree network, suggesting a certain correlation between haplotype clusters and geographical distribution. Further experiment should be done for Liaoning strains due to the small sample size and number of single haplotype.

### 3.3. Population Genetic Structure

The MEGA6 software package was used to calculate the genetic distances of different geographical strains (Table 2). Genetic distance is one of the indicators to measure the genetic diversity within a population. It can be seen from the table that the genetic distance within each geographical population is ordered as Fujian > Yunnan > Hainan > Taiwan > Guangdong > Liaoning. The genetic distance between populations ranges from 0.00091 to 0.02473 with an average of 0.01925. The genetic distance between Fujian and Yunnan strains is largest. The genetic distances between Fujian and other geographical strains are more than 0.02, indicating the significant difference between Fujian and other regions. The genetic distance between Liaoning and Guangdong strains is the smallest.

Arlequin 3.1 was used to calculate the genetic differentiation coefficients (Fst) among geographical strains and the results were summarized in Table 3. Fst is an indicator to measure the genetic differentiation between populations. Great Fst value means greater genetic differentiation and less gene exchange. When Nm is greater than 1, gene exchange can prevent genetic differentiation between populations caused by genetic drift. The Nm values between Fujian, Guangdong, and Taiwan strains are all greater than 1, indicating frequent genetic exchanges between these geographical populations. The Fst value (0.11363) and Nm value (1.95012) between Hainan and Yunnan strains indicate the smallest genetic differentiation and frequent gene exchange between them. However, the Nm values of the two geographical strains with other geographical strains are less than 1, indicating limited genetic exchanges.

The AMOVA analysis (Table 4) shows that the variation within populations (78.13%) is greater than that between populations (21.87%), which suggests that the genetic differentiation of* Aedes albopictus* population structure mainly comes from the population interior. The total of Fst value (0.21871) and Nm value (0.893) indicates that the overall gene flow of* Aedes albopictus* failed to prevent the population differentiation caused by genetic drift and there is a certain level of genetic differentiation in the population.

### 3.4. Group Dynamics

We used the mismatch analysis of Arlequin (Table 5) to assess the reliability of expansion through SSD and HR parameters. If the difference between the two parameters is not statistically significant (i.e., P > 0.05), the assumption of population expansion could not be rejected, which is in line with the original group expansion hypothesis. Overall analysis from* Aedes albopictus* indicates that the p-values of SSD and HR are all greater than 0.05, suggesting an expansion of* Aedes albopictus* in history. From the perspective of geographical strains, the p-values of SSD and HR of Hainan, Yunnan, and Taiwan strains are all greater than 0.05, suggesting an expansion of* Aedes albopictus* in these areas in history. In Fujian and Guangdong, only p-value of HR parameter is greater than 0.05, suggesting that* Aedes albopictus* in these areas do not have significant expansion in history.

**Figure 4:**
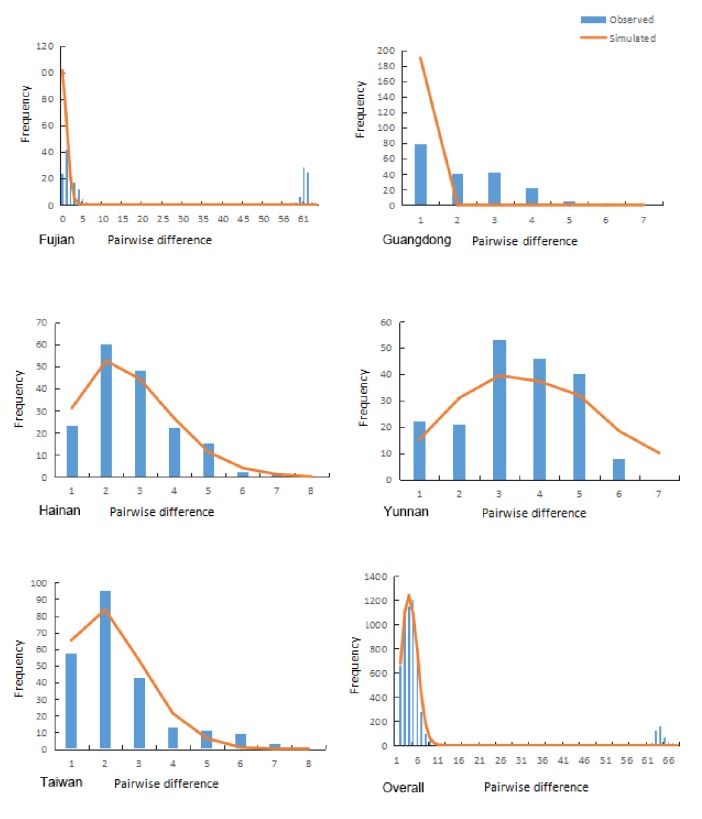
Mismatch distribution of* Aedes albopictus MT-COI* gene from different geographical regions.

We mapped the bifurcation distribution of different geographical strains of* Aedes albopictus MT-COI *gene (Figure 4) and observed a fitting between the expected and the observed values, which also indicates population expansion. Generally speaking, the population is suggested to be in balance when the observed values of bifurcation distribution do not coincide with the expected ones (i.e., the figure will show multipeaks); otherwise (i.e., single peak) it will indicate a population expansion [32]. Specifically, the observed values of Hainan, Yunnan, and Taiwan strains coincide with the expected values and showed a single peak distribution, but the distributions of Fujian and Guangdong strains are not consistent. The overall bifurcation distribution of* Aedes albopictus* presents a single peak distribution, indicating population expansion.

Tajima's D value and Fu's Fs value (Table 6) were calculated using the neutrality test of the Arlequin software (Table 6). In theory, negative Tajima'D and Fu's Fs values will indicate a population expansion in history. In our case, the* D* value (−1.991) and Fs value (−24.983) of overall population both reach a significant level, indicating a significant population expansion in history. As for specific geographical strains, except for Fujian strains with positive D value and Yunnan strains without significant negative D value, other geographical strains are with statistically significant negative D and Fs values. The general results are consistent with the mismatch analysis.

According to the formula *θ* = 2Nu, the effective size of female mosquito population was estimated based on *θ*_0_ and *θ*_1_ before and after expansion. When *θ*_0_ = 0.443 and *θ*_1_ = 99999, the effective number of female mosquitos in the population is estimated to be 3.4 × 10^4^~7.6 × 10^9^. According to *τ* (95% CI) = 2ut = 3.943, the population expansion occurred in 3.0 × 10^5^ years ago.

### 3.5. The Relationship between Genetic Differentiation and Geographical Distance

**Figure 5:**
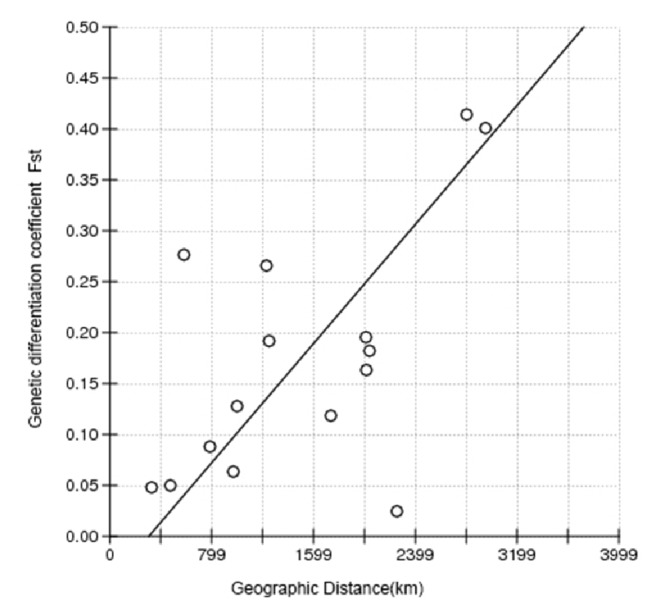
The relationship between genetic differentiation of* Aedes albopictus MT-COI *gene and geographical distance.

The Mantel test was performed on the geographical distances between different collection points and* Aedes albopictus MT-COI* gene sequences. The correlation curve between genetic differentiation coefficients Fst and geographical distances was illustrated in Figure 5. The correlation between population Fst and geographical distances (r = 0.5789, p = 0.0120) was statistically significant, indicating a positive correlation between genetic differentiation and geographical distance.

## 4. Discussion

The genetic diversity of insect species can arise from two sources: internal and external causes. The internal causes are genetic mutation and adaptability of insect itself while the external factors are closely related to ecological environment of insects. The accumulation of genetic difference will lead to reproductive isolation and thus the formation of new species. A few scholars have studied the diversity of* Aedes albopictus* by different methods. Preliminary studies have confirmed that there is a certain degree of genetic differentiation among* Aedes albopictus* populations. Therefore, further studies on genetic variation of* Aedes albopictus* population are needed to provide valuable information for the prevention and control of dengue fever. Also, new mosquito species can become the vector of new diseases, and it is critical to surveil the mosquito species in a geographical region.

Haplotype diversity (Hd) and nucleotide diversity (Pi) are two important indicators to measure the diversity of a species population [33]. The haplotype diversity and nucleotide diversity of* Aedes albopictus* in this study (Hd = 0.882, Pi = 0.01017) are higher than those in* Aedes aegypti* (Hd = 0.740 ± 0.017, P = 0.0065 ± 0.0003), indicating high diversity* of Aedes albopictus* population.* Aedes albopictus* exhibited high Hd and low Pi mode probably because of rapid population expansion after the bottleneck effect. The formation of a new haplotype is due to a single base variation, which has less effect on nucleotide diversity, so increasing nucleotide diversity requires more time to accumulate than increasing haplotype diversity. The bottleneck effect can eliminate the accumulation of nucleotide diversity in the past but can also cause mutations at the base site, resulting in a pattern of high Hd and low Pi. The possible reasons for the bottleneck of* Aedes albopictus* may be as follows: on the one hand, in terms of neutral test of* Aedes albopictus* (D = −1.99, P = 0.002; Fs = −24.98, P = 0.000) and the bifurcation distribution representing a single peak,* Aedes albopictus* has been or is in a state of population expansion. On the other hand, dengue fever has caused great harm to human health since it was recognized that controlling the number of media mosquitos is critical for controlling the spread of the disease. Therefore, a wide range of remediation media mosquitos have a greater impact on* Aedes albopictus* population.

In this study, the genetic distances between different geographical populations ranged from 0.00091 to 0.02473, which are higher than those of* Aedes albopictus* in different regions of Guangzhou about 0.000~0.007 in a previous study. Among them the genetic distances between Fujian and other geographical strains were greater than 0.02 (Table 2), which is greater than the difference of less than 2% of the genetic distance within 98% of the species proposed by Hebert [10], indicating a large genetic difference between Fujian* Aedes albopictus* and other geographical strains. Haplotype diversity, nucleotide diversity, and genetic distance are all indicators to reflect the genetic diversity of population. The results of the three indicators in this study (Tables 1 and 2) basically showed the largest value of Fujian strains, indicating Fujian population with the largest genetic diversity. In the pedigree network diagram, Haplotypes h2 is found to be a shared haplotype among Fujian, Guangdong, Taiwan, and Liaoning and developed into other haplotypes through direct or indirect evolution of 1~5 steps. However, the NJ tree shows that 3 haplotypes (h4, h11, and h12) of Fujian clustered independently into one category with high confidence, which further indicates the result that Fujian strains of* Aedes albopictus* have obvious genetic structure changes and also verified early studies. Those studies suggested that there is a certain degree of genetic differentiation of different geographical strains of* Aedes albopictus* in Fujian province. Due to the farthest flight distance of* Aedes albopictus* not exceeding 500 meters, it is very difficult to invade from the exterior in short time. The reason for this phenomenon may be related to the ecological and climatic environment of* Aedes albopictus* in Fujian. Fujian is located in a subtropical maritime monsoon climate, whose complex topography forms a variety of local climate, thus forming different ecological environments, which provide favorable conditions for breeding and living* Aedes albopictus*. Fujian geographical strains of* Aedes albopictus* haplotypes diversity, the causes of specific haplotype, and whether there is a special significance in the mosquito-borne disease transmission need further study.

Morton [34] believed that when the gene flow Nm value is greater than 1, it means that the gene exchange is frequent, which can prevent the interpopulation differentiation caused by genetic drift. When the value is less than 1, it indicates that gene exchange is blocked. In this study, the* Aedes albopictus* Nm value is less than 1 (Nm = 0.893), indicating that the level of gene exchange failed to prevent population differentiation caused by genetic drift and there is a certain level of genetic differentiation between populations. In terms of Nm value of different geographical strains (Table 3), the Nm value between Hainan and Yunnan strains is greater than 1, indicating that* Aedes albopictus* in the two places had frequent genetic exchange. However, the Nm values between (Yunnan + Hainan strains) and Guangdong, Taiwan and Liaoning strains are less than 1, indicating that* Aedes albopictus* gene exchanges between (Yunnan + Hainan) and the four regions are hindered. This is consistent with NJ tree that Yunnan + Hainan was clustered into one category. The NJ tree and gene flow show that the genetic exchanges frequently happen among Taiwan, Fujian, and Guangdong mosquitos, suggesting that Taiwan strains have the same type of geographical populations as Fujian and Guangdong strains. No relevant reports have been found yet.

Finally, this study shows that the genetic differences within* Aedes albopictus* population are larger than those between populations. Due to the different ecological and climatic conditions in different regions, there are various kinds of natural barriers. However,* Aedes albopictus* belongs to the semihabitat mosquito and is closely related to human activities, the geographical barrier failed to completely prevent the gene flow, so genetic differentiation mainly which comes from internal and genetic differentiation among the populations is not high. The result of the Mantel test (r = 0.5789, p = 0.0120) shows that the degree of CO I gene sequence variation is positively correlated with geographical distance. Further experiments will be conducted to confirm this conclusion. Giving more and more genetic sequences of mosquitos in different regions has been published; it would be interesting to study the genetic differences of mosquito populations throughout China or across a continent in the future.

## 5. Conclusion

In conclusion, our results suggest that (1) there are genetic differences among* Aedes albopictus* populations in different geographical regions; (2) there is a positive correlation between the genetic differences of* Aedes albopictus* population and their geographical distances; and (3) DNA barcoding of* MT-COI* gene is an effective method to study the genetic structure of* Aedes albopictus*.

## Figures and Tables

**Table 1:** Haplotype diversity and nucleotide diversity of different geographical strains.

**Collecting ** **places**	**Number of samples**	**Number of haplotypes**	**Haplotype type (n)**	**Polymorphism sites (s)**	**Haplotype diversity (Hd)**	**Nucleotide diversity (Pi)**
Fujian	20	12	h1(2),h2*∗*(7),h3(1),h4(2),h5(1), h6(1),h7(1),h8(1),h9(1),h10(1), h11(1),h12(1)	66	0.87895	0.03316
Guangdong	20	7	h2*∗*(13),h6*∗*(1),h13(1),h14(1), h15(1),h16(1)h17(2)	8	0.58421	0.00173
Hainan	19	9	h18*∗*(5),h19*∗*(5),h20(1),h21(1), h22(3),h23(1),h24(1),h25(1), h26(1),	11	0.86550	0.00265
Yunnan	20	11	h18*∗*(4),h19*∗*(1),h27(1),h28(6), h29(1),h30(1),h31(1),h32(1), h33(1),h34(2),h35(1)	13	0.88421	0.00372
Taiwan	22	8	h2*∗*(9),h36(1),h37(7),h38(1), h39(1),h40(1),h41(1),h42(1)	11	0.75325	0.00215
Liaoning	5	1	h2*∗*(5)	0	0.00000	0.00000

*Note*. *∗* denotes shared haplotypes.

**Table 2:** The genetic distances within and between different geographical strains.

**Collecting places**	**The genetic distances within populations**	**The genetic distances between populations**				
		**Fujian**	**Guangdong**	**Hainan**	**Yunnan**	**Taiwan**
Fujian	0.03535					
Guangdong	0.00173	0.02187				
Hainan	0.00266	0.02403	0.00421			
Yunnan	0.00374	0.02473	0.00466	0.00361		
Taiwan	0.00216	0.02252	0.00223	0.00462	0.00504	
Liaoning	0.00000	0.02122	0.00091	0.00337	0.00374	0.00132

**Table 3:** The Fst (lower triangle) and Nm (upper triangle) values of different geographical strains of *Aedes albopictus  MT-COI* gene.

**Collecting places**	**Fst∖Nm**					
	**Fujian**	**Guangdong**	**Hainan**	**Yunnan**	**Taiwan**	**Liaoning**
Fujian		1.40596	0.96993	0.94554	1.17849	9.35430
Guangdong	0.15097		0.27134	0.35618	1.71757	−4.09084
Hainan	0.20493	0.47953		1.95012	0.27040	0.29583
Yunnan	0.20911	0.41242	0.11363		0.34867	0.48844
Taiwan	0.17501	0.12706	0.48040	0.41759		7.06422
Liaoning	0.02603^*∗*^	−0.06509^*∗*^	0.45802	0.33855	0.03418^*∗*^	

*Note*. *∗* represents p>0.05.

**Table 4:** The AMOVA analysis of different geographical strains of *Aedes albopictus MT-COI* gene.

**Source of variation**	**Degree of freedom**	**Variation components**	**Percentage of variation (%)**
Between populations	5	0.76116	21.87
Within population	100	2.71898	78.13

*Note*. Fst: 0.21871, Nm: 0.893.

**Table 5:** The mismatch analysis of different geographical strains of *Aedes albopictus*.

	**Overall**	**Fujian**	**Guangdong**	**Hainan**	**Yunnan**	**Taiwan**	**Liaoning**
SSD	0.520	0.00^*∗*^	0.00^*∗*^	0.50	0.20	0.45	0.00^*∗*^
HR	0.790	1.00	1.00	0.25	0.30	0.40	0.00^*∗*^

*Note*. *∗* represents P<0.05.

**Table 6:** The neutrality test of different geographical strains.

**Collecting places**	**Tajima's *D***		**Fu's *Fs***		**Tau (95%)**
	***D *value**	**P value**	***FS* value**	**P value**	
Overall	−1.991	0.002	−24.983	0.000	3.943
Fujian	0.703	0.280^*∗*^	−6.878	0.003	1.008
Guangdong	−1.672	0.037	−29.191	0.000	0.000
Hainan	−1.604	0.038	−27.472	0.000	2.242
Yunnan	−1.205	0.119^*∗*^	−26.629	0.000	3.820
Taiwan	−1.837	0.019	−27.981	0.000	2.070
Liaoning	0.000	-	*∞*	-	0.000

Note: ^*∗*^P>0.05.

## Supplementary Materials

Supplementary Materials
** Table S1: variable sites in* MT-COI* haplotype sequences of* Aedes albopictus* in different geographical regions.** The dot “.” denotes consensus nucleotide with coI h1.
