# Supplementary material for: Genetic Polymorphism Study on Aedes albopictus of Different Geographical Regions Based on DNA Barcoding
Source: Biomed Res Int. 2018 May 29;2018:1501430. doi: 10.1155/2018/1501430 (PMC5996416; doi:10.1155/2018/1501430)
Supplement: Supplementary Materials — Table S1: variable sites in MT-COI haplotype sequences of Aedes albopictus in different geographical regions. The dot “.” denotes consensus nucleotide with coI h1. [file 1501430.f1.docx]

**Table S1.** Variable sites in *MT-COI* haplotype sequences of *aedes albopictus* in different geographical regions. The dot “.” denotes consensus nucleotide with coI h1.

| 111111111 1112222222 2233333333 3333444444 4444445555 5555555555 5556666666 |
| --- |
| 112233455 5566777888 8003334667 7990156677 8812223444 6677011222 3579990111 2444555568 8990123444 |
| 1092318004 6859029256 8390796391 2375296814 6932584036 1403128149 3183698147 0478036826 9264681136 |
| coⅠh1 ACCTATGCCG GTCTTAATTG TTATGTAATT CCCCCTTACC AGCAAGGTTT ATTGTGCATT AATAATGAAA ATTCACATCA CATATGATAT |
| coⅠh2 .......... .......... .......... .......... G......... .......... .......... .......... .......... |
| coⅠh3 .......... .......... .......... .......... G......... .......... ........G. .......... .......... |
| coⅠh4 .TTAGG.AT. A..CA.CCA. A..A.A.TA. TTTTT.CTTT TTT.TATC.. T.C..ATTA. .GATTAA.TT TA.T.TT.GT ...TCA.CTC |
| coⅠh5 .......... .......... .......... .......... .....A.... .......... .......... .......... .......... |
| coⅠh6 ..T....... .......... .......... .......... G......... .......... .......... .......... .......... |
| coⅠh7 .......... .......... .......... .......... G....A.... .......... .......... .......... .......... |
| coⅠh8 ..T..C.... ...C....A. .......... .......... G......... .......... .......... .......... .......... |
| coⅠh9 ......A... .......... .......... .......... G....A.... .......... .......... .......... .......... |
| coⅠh10......A... .....G.... .......... .......... G....A.... .......... .......... .......... .......... |
| coⅠh11.TTAGG.AT. A..CA.CCA. A..A.A.TA. TTTTTCCTTT TTT.TATC.. T.C..ATTA. .GATTAA.TT TA.T.TTCGT ...T.A.CTC |
| coⅠh12.TTAGG.AT. A..CA.CCA. A..A.A.TA. TTTTT.CTTT TTT.TATC.. TCC..ATTA. .GATTAA.TT TA.T.TT.GT ...TCA.CTC |
| coⅠh13.......... .......... .......... .......... G......... .......... .......... .......... .G........ |
| coⅠh14.......... .........A .C........ .......... G....A.... .......... .......... .......... .......... |
| coⅠh15......A... A......... .......... .......... G......... .......... .......... .......... .......... |
| coⅠh16.......... .......... .......... .......... G......... .......... .......... .......... ..C....... |
| coⅠh17.......... .......... .C........ .......... G....A.... .......... .......... .......... .......... |
| coⅠh18.......... .......... .......... .......... G........C .......... .......... .......... .......... |
| coⅠh19.......... .......... .......... .......... G........C .......... .......... .......... .....A.... |
| coⅠh20.......... .......... .......... .......... G.......CC .......... .......... .......... .....A.... |
| coⅠh21.......... .......... .......... .......... G........C ....C..... .......... .......... .......... |
| coⅠh22.......... .......... .......... .......... G....A...C .......... .......... .......... .....A.... |
| coⅠh23.......... .......... .......... .......... G........C .C........ .......... .......... .....A.... |
| coⅠh24.......... .......C.. .......... .......... G........C .......... .......... .......... .......... |
| coⅠh25.......... .......... .......... .......... G........C .......... .......... ..C....... ......G... |
| coⅠh26......A... .......... ..G....... .......... G........C ...A...... .......... .......... .....A.... |
| coⅠh27.........A .......... .......... .......... G........C .......... .......... .......... .....A.... |
| coⅠh28G......... .......... .......... .......... G........C .......... .......... .......... T......... |
| coⅠh29.......... .......... ....A..... .......... G........C .......... .......... .......... .......... |
| coⅠh30.......... ........C. .......... .......... G........C .......... .......... .......... .......... |
| coⅠh31.......... .......... .......... .......... G........C .......... G......... .......... .......... |
| coⅠh32....T..... .......... .......... .......... .........C .......... .......... .......... .......... |
| coⅠh33.......... .......... .......... .......... G........C ....C..... .......... ....G..... .......... |
| coⅠh34.......... .......... .......... .......... G........C .......... ......A... .......... .....A.... |
| coⅠh35.......... ..T....... .......... .......... G........C .......... ......A... .......... .....A.... |
| coⅠh36.......... .......... ......G... .......... G......... ...A...... .......... .......... .......... |
| coⅠh37.......... .......... .......... .......... G......... ...A...... .......... .......... .......... |
| coⅠh38.......... .......... .......... .......... G......... ...A.....C .......... .......... .......... |
| coⅠh39.......... .......... .......... .......... G......... .......... ...T..AG.. ..C....... .......... |
| coⅠh40.......... .......... .......... .......... G......... .......... G......... .......... .......... |
| coⅠh41.......... .C........ .......... .......... G..G...... .......... .......... .......... .......... |
| coⅠh42.......... .......... .........C .......... G......... .......... .......... .......... .......... |
